# Design of transformation initiatives implementing organisational agility: an empirical study

**DOI:** 10.1007/s43546-021-00073-6

**Published:** 2021-04-30

**Authors:** Ivan Kovynyov, Axel Buerck, Ralf Mikut

**Affiliations:** 1 Imperial College Business School, London, UK; 2 Kobaltblau Management Consultants GmbH, Munich, Germany; 3 Institute for Automation and Applied Informatics, Karlsruhe Institute of Technology, Karlsruhe, Germany

**Keywords:** Agile, Agile organisations, Organisational design, Organisational agility, Agile transformation

## Abstract

This study uses 125 responses from companies of all sizes predominantly headquartered in Germany, Switzerland, France and UK to reveal perceptions of the drivers of organisational agility. It further investigates current understanding of managing principles of multiple organisational dimensions such as culture, values, leadership, organisational structure, processes and others to achieve greater organisational agility. The data set is disaggregated into four major profiles of agile organisations: laggards, execution specialists, experimenters, and leaders. The approach to agile transformation is analysed by each of those profiles. While the positive effect from a more holistic approach is confirmed, leaders tend to focus more on processes and products rather than project work. Respondents perceive that IT, product development and research are most agile functions within their organisations, while human resources, finance and administration are considered being not agile. Furthermore, organisations with higher levels of organisational agility tend to use more than one agile scaling framework. Implications on theories of agile transformations and organisational design are discussed.

## Introduction

Organisations aspire achieving greater agility, most often defined as the ability to fluidly react to changes in customer behaviour and market conditions (Overby et al. 2006; Keller et al. 2019; Wendler 2013). Senior executives direct attention to implementing agile practices within their organisations to improve its strategic positioning (Kotter 2012), improve decision making (Rigby et al. 2020), and facilitate exploration of new avenues of revenue (Ghezzi and Cavallo 2020). Since rapid adaptation and agility improve performance in volatile environments (Rafique et al. 2018; Drury-Grogan 2014), it is important to examine what decision makers currently perceive as enabling factors of organisational agility and what are ways to achieve it.

Both agile software development and agile organisational design are theorised as influencing organisational agility, but these theoretical streams have evolved as independent literature. In many frameworks, organisational agility arises from cultural changes, strategic flexibility and managerial practices (Wendler 2013; Kalenda et al. 2018). Further studies find that agile architectures (Leffingwell et al. 2008), ways of working (Lindsjørn 2016) and employee empowerment (Menon 2001) improve the organisation’s ability to respond rapidly and effectively. Studies on organisational design, however, attribute agility to structures that facilitate flexibility and impose changes in managerial control systems (Bernstein et al. 2016; Kotter 2012).

Gaps nevertheless exist in understanding how companies attain organisational agility. First, it is unclear whether organisational agility comes from technical excellence or agile organisational design. Second, models examine organisational agility independent of organisation-wide transformation efforts (Pries-Heje and Mathiassen 2006; Ambrose and Morello 2004). Third, although studies have generated interesting results on designing individual dimensions of organisational agility such as structure, leadership style or software development, there is a paucity of global, multi-dimensional studies addressing interdependence across individual dimensions. Also, it lacks an empirically backed overview of best practices for design and implementation of agile transformation initiatives.

This study uses 125 responses from companies of all sizes headquartered in Germany, Switzerland, France and UK to reveal perceptions of the drivers of organisational agility. It further investigates current understanding of managing principles of multiple organisational dimensions such as culture, values, leadership, organisational structure, processes and others to achieve greater organisational agility. Our study is the first empirical effort to address three questions about designing and implementing agile transformation initiatives: What are major profiles for agile organisations? How do organisations balance efforts across individual organisational dimensions, such as structure, leadership style, culture, software development practices and project work? What are best practices in designing agile transformation initiatives?

The data set is disaggregated into four major profiles of agile organisations: laggards, execution specialists, experimenters, and leaders. The approach to agile transformation is analysed by each of those profiles. While the positive effect from a more holistic approach is confirmed, leaders tend to focus more on processes and products rather than project work. Respondents perceive that IT, product development and research are most agile functions within their organisations, while human resources, finance and administration are considered being not agile. Furthermore, organisations with higher levels of organisational agility tend use more than one agile scaling framework. These findings inform an important area of managerial practices and present opportunities for future research.

## Theory

### Literature review and research questions

### Technical excellence vs. agile organisational design

Agility involves organisation’s responsiveness to changes (Overby et al. 2006) and a proactive rather than reactive attitude. Early studies of agility relied on observations from self-governed, autonomous software engineering teams (Reich 1999) and from process improvements within manufacturing systems (Vokurka and Fliedner 1998; Sharifi and Zhang 1999; Takeuchi and Nonaka 1998). However, more recent studies refer to agility not only as an outcome of technological achievement but rather as a result of human ability, skills and motivation (Sherehiy et al. 2007). Shifting this understanding from rather a technological implementation to an enterprise management system has reframed agility as an organisational agility.

A well-examined characteristic associated with organisational agility is agile organisational design. Novel organisational forms^1^ facilitating value orientation and cross-functional work arise: value streams (Rother and Shook 2003), Holacracy (Robertson 2015; Bernstein et al. 2016), DevOps (Ebert et al. 2016) and others. Similarly, new structures require new roles and responsibilities: agile coach (Davies and Sedley 2009) and product owner (Bass 2015). Agile scaling frameworks^2^ lay foundations for implementing agility on an organisation-wide level: SAFe (Leffingwell 2018), LeSS (Larman and Vodde 2016), Spotify model (Kniberg and Ivarsson 2012), Scrum of Scrums (Sutherland 2005) and others.

From a technical perspective, organisational agility is determined by agile software development practices (Beck et al. 2001; Martin 2002). Studies suggest that frequent delivery, small batch size, agile requirements engineering and agile testing procedures are essential technical antecedents for organisational agility (Chow and Cao 2008; de Souza Bermejo et al. 2014). Furthermore, team diversity and autonomy are crucial for success in agile teams (Gwanhoo and Weidong 2010; Lindsjørn 2016).

However, it is unclear whether organisational agility comes from agile organisational design alone or it requires appropriate technical excellence. Also, there is a paucity of in-depth analyses of mechanisms imposing organisational agility through technical improvements and organisational changes.

Recent studies suggest that organisational agility arises from corporate values, technology, change management practices, agile collaboration styles and structures (Wendler 2012). It appears to have a more subtle relationship to individual organisational dimensions; therefore, we investigate how organisations balance efforts across those dimensions to thrive organisational agility.

Finally, some scholars argue that agility supports generating value from digital technologies (Kovynyov and Mikut 2019; Ghezzi and Cavallo 2020). Therefore, we briefly elaborate on this argument by investigating the relationship between organisational agility and digital initiatives.

### Scaling agile

Previous studies suggest that many benefits of organisational agility derive from scaling agile practices (Kalenda et al. 2018). Therefore, the demand for agile scaling frameworks has increased (Rigby et al. 2016). Some papers investigate scaling agile methods in large software development projects (Mashal and Rozilawati 2016), other focus on using agile methods in large-scale product development initiatives (Petri and Maarit 2008). Increase in the agile scaling effort creates need for the selection criteria (Diebold et al. 2018).

In line with prior research, we refer to agile scaling frameworks as conceptual frameworks implementing agile values, principles and practices on the enterprise-wide level, for instance, scaled agile framework SAFe (Leffingwell 2018), Spotify model (Kniberg and Ivarsson 2012), Scrum of Scrums (Sutherland 2005) and others. We expect a positive relationship between using agile scaling frameworks and the level of organisational agility and address the current usage of frameworks in corporate environments.

### Agile transformation initiatives and measuring agility

Research has assessed challenges and success factors of agile transformation initiatives (Dikert et al. 2016). Although agile transformation design has been studied in context of large-scale software development projects (Mashal and Rozilawati 2016) and product development initiatives (Petri and Maarit 2008), there is less empirical research in context of companies seeking agility on the organisation-wide level. Recent studies suggest complex relationships between individual organisational dimensions when implementing and measuring agile transformation effort (Petri and Maarit 2008). Several maturity models are used to understand and measure those relationships (Wendler 2013; Gren et al. 2015).

In line with prior research, we expect that individual organisational dimensions are affected by agile transformations in different ways and investigate this relationship by identifying major profiles of agile organisations. Finally, we consider the design of agile transformation initiatives and derive best practices when designing and implementing such initiatives.

### Suggested framework

Research confirms impact of agile practices on multiple organisational dimensions (Sherehiy et al. 2007; Wendler 2013). Some scholars suggest that organisational agility impacts corporate values, technology, change management practices, collaboration styles and organisational structure (Wendler 2012). Other studies draw relationship to architecture (Leffingwell et al. 2008), ways of working (Lindsjørn 2016) and people management (Menon 2001). Furthermore, studies on organisational design attribute agility to structures that facilitate flexibility and impose changes in managerial control systems (Bernstein et al. 2016; Kotter 2012).

We suggest, therefore, the following six organisational dimensions to assess the impact of organisational agility on organisations:

- culture, values and leadership,
- organisation and structure,
- delivery and software development,
- product development,
- ways of working,
- enterprise architecture.

*Culture, values and leadership* cover leadership and operating styles of the management, norms and behaviours people follow across the organisation, how people interact at work with each other within the organisation and with external partners such as clients and vendors (Bradach 1996). Related agile practices and tools include agile goal setting using Objectives & Key Results method (OKR) (Niven and Lamorte 2016), agile leadership practices (Baker and Thomas 2007), continuous improvement with Kaizen (Anders 1997), feedback culture (Strode et al 2009), employee empowerment (Menon 2001), self-organisation, Management 3.0 practices (Appelo 2016), agile mindset, fail-faster-principle, agile coaching (Davies and Sedley 2009) and others. Key differentiator between agile and non-agile organisations for this domain is the attitude towards risk-taking. Agile organisations consider failure as an essential part of learning and embrace taking calculated risks, while traditional organisations usually follow plan-and-execute approaches cultivating zero-failure-tolerance (Strode et al 2009).

*Organisation and structure* refer to ways “in which tasks and people are specialised and divided, and authority is distributed” across the organisation (Bradach 1996). This dimension includes grouping of activities and reporting relationships into organisational units, formal and informal procedures and processes used to manage the organisation. Related agile practices and tools are cross-functional teams (Parker 2003), new agile roles (agile coaches (Davies and Sedley 2009), product owners (Bass 2015 and others), novel organisational forms (value streams (Rother and Shook 2003), Holacracy (Robertson 2015; Bernstein et al. 2016), DevOps (Ebert et al. 2016) and others), and agile scaling frameworks SAFe (Leffingwell 2018), LeSS (Larman and Vodde 2016), Spotify model (Kniberg and Ivarsson 2012) and others). With that in mind, the level of cross-functional collaboration can be seen as the major differentiator between agile and non-agile organisations. In agile organisations, individual functional parts collaborate seamlessly across divisions of an organisation to create value. General work is organised in a cross-functional manner rather than in functional silos. The collaboration mode reconfigures fluidly and adapts to the changing environment. Traditional Tayloristic organisations, however, exhibit behaviours where individual functional parts follow their own agendas and focus on local optima rather than improving the entire system.

*Delivery and software development* include all activities associated with implementing new software solutions within the organisation such as software development life cycle, project management approach and maintenance procedures. This domain focuses in particular on ways of organising large-scale software projects such as introduction of an application, relaunch or replacement of existing systems and applications. Here, organisations tend to use agile practices and tools such as extreme programming (Beck et al. 2001), disciplined agile delivery (Ambler and Lines 2012), test-driven development (Beck 2003), test automation (Figueiredo et al. 2012), continuous delivery (Humble and Farley 2010), pair programming (Vanhanen and Korpi 2007), minimal viable products (Lenarduzzi and Taibi 2016), minimal marketable features (Cleland-Huang and Denne 2005) and others. Considering the project work, the amount of up-front planning can be considered as the key differentiator. While traditional organisations spend significant amount of work for high-level planning activities before the project kick-off, agile organisations distribute those activities over the entire project duration. The batch size (amount and magnitude of software changes in one release cycle) and release frequency can be seen as key differentiators between agile and non-agile organisations. Agile organisations tend to use processes and procedures allowing them to release small pieces of software frequently. Traditional organisations usually follow a more fixed schedule of major monthly or quarterly releases.

*Product development* refers to creation and launch of new products that satisfy a newly created customer need or market niche. This dimension also covers modification of existing products. Related agile practices include customer journey mapping, design thinking (Liedtka 2018), customer centricity (Shah 2006), design sprints (Richard et al. 2015), Lean Start-up methodology (Reis 2011) and others. Distance to the customer is the key differentiator (Rigby et al. 2016). Agile organisations are able to capture changes in customer needs and fluidly reconfigure.

*Ways of working* cover practices of organising, performing, leading, along with new approaches to recruiting, developing and engaging employees. Ways of working usually refer to Scrum (Schwaber and Beedle 2002), Kanban (Ahmad et al. 2013), Kaizen (Anders 1997), agile retrospectives (Derby and Larsen 2006), Beyond Budgeting (Libby and Murray 2010) and others. Levels of employee autonomy and amount of decision rights a regular employee is equipped with can be considered as differentiating factors in this domain.

*Enterprise architecture* refers to the fundamental orchestration of software systems and its components, their relationship to each other and towards the external environment, as well as general principles of governance relating to design and evolution of those systems (Winter and Fischer 2006). This organisational dimension further includes a set of values, principles and practices that support active, evolutionary design and architecture of the organisation’s systems landscape. Here, organisations use such practices as architectural runway (Buchmann et al. 2012), agile architecture (Leffingwell et al. 2008) and others. Agile architectures are more federated (Leffingwell et al. 2008). Organisations are seeking to solve a trade-off between organisational agility and reliability through decoupling (Keller et al. 2019). They have loosely coupled, independent modules, while traditional architectures are predominantly build from monolithic legacy systems.

## Data and method

### Measures

This subsection aims to explain the origin and calculation of key dimensional measures used in the data analysis.

In our analysis, we use the following dimensional measures, as suggested in the "Suggested framework":

- D1: Culture, values and leadership
- D2: Organisation and structure
- D3: Delivery and software development
- D4: Product development
- D5: Ways of working
- D6: Enterprise architecture

These measures are difficult to quantify; therefore, we reviewed recent papers and collected qualifying questions for each dimension (Gren et al. 2015; Gunsberg et al. 2018; Rigby et al. 2020). The responses to these questions are mapped into scores. The score ranges from 1 to 5 with 1 being the lowest and 5 being the highest scores. The scores represent the maturity levels and are defined as follows:

1. non-agile,
2. selected basic agile principles and tools are implemented,
3. core agile principles and tools are implemented,
4. advanced agile principles and tools are implemented,
5. front-running, novel agile tools and practices are piloted.

We used the following formula to calculate the dimensional score:

1 $$\begin{aligned} D_i = \frac{1}{2} \cdot \left( \frac{1}{\# M_i} \sum _{m \in M_i} Q_m + \underset{m \in M_i}{\min } Q_m \right) \end{aligned}$$

where

- *i* = dimensional measure, $$i \in \{1...6\}$$,
- $M_i$ = set of the questions from the questionnaire related to the dimension *i*,
- $Q_m$ = the answer to the question m.

Equation (1) builds upon the assumption that the pure mean value of all answers does not sufficiently reflect the level of organisational agility. The answers pointing out to the lowest agility levels often reveal the true level of the agile maturity. Therefore, we explicitly included the minimum value of the answers into Equation (1). With this in mind, the answer with the lowest value has a direct influence on the dimensional score supporting the idea that bottlenecks should be addressed first to reach a higher maturity level.

The M-sets are defined as follows (the numeration is based on the questions as listed in "Survey questions"):

- $$M_1 = \{ 5a, 5b, 5c, 5d \}$$
- $$M_2 = \{ 5e, 5f, 5g \}$$
- $$M_3 = \{ 7a, 7b \}$$
- $$M_4 = \{ 7c \}$$
- $$M_5 = \{ 7d, 7e, 7f \}$$
- $$M_6 = \{ 7g, 7h \}$$

$Q_m$ is defined as follows:

- $Q_m = 1$ for strongly disagree, definitely false
- $Q_m = 2$ for somewhat disagree, probably false
- $Q_m = 3$ for neither agree nor disagree, neither true of false
- $Q_m = 4$ for somewhat agree, probably true
- $Q_m = 5$ for strongly agree, definitely true

### Target group and distribution

The target group consists of senior executives, business leaders and agile practitioners in small, medium-size and large enterprises predominantly in Europe, regardless of industry or particular business area. Also, the target group includes organisations which have recently undertaken an agile transformation. The respondents should have gained experience in applying agile principles and practices across their organisations in the recent past, for instance, through participation in agile transformation programmes as a sponsor, agile coach, change manager, line manager or senior executive. Respondents can be part of internal IT organisation, but also work for the business units. We worked with kobaltblau Management Consultants to compile the contact list that fits the selected profile. Candidates have been randomly selected from the provided list. The survey took place in April - May 2019.

It was a voluntary survey and was conducted by means of a digital questionnaire (cf. "Survey questions"). We created questions in three languages: English, German, and French. Participants could switch across the languages at any point in time during the survey.

A number of measures has been implemented to prevent survey taking fatigue:

- one question at a time appeared on screen,
- questions numbers were hided,
- the overall progress bar was set up to indicate percentage completed.

Table 1 reports a breakdown of the data sample response rate by distribution type. We approached the audience of 1044 persons and obtained 210 responses. 85 out of 210 responses appeared to be partial responses (participants have not finished answering the questionnaire) and were excluded from further consideration. The final sample data set included, therefore, 125 responses. Higher response rates were obtained from individual invites over email. The response rate of individual invites was 33%, while only 3% of mass mail receivers responded to the invitation. Same for the completion rate: 61% of the participants that have been approached individually and responded to the invitation have completed the survey, while the completion rate for the mass mail receivers was only 40%.

**Table 1:** Key response statistics by distribution type

	Mass mailing	Invite over email	Total
Audience size	444	600^∗^	1044
Total responses, thereof	13	197	210
Partial responses	8	77	85
Finished responses	5	120	125
Response rate	3%	33%	
Completion rate	40%	61%	

^∗^Estimate

### Respondent profile

Table 2 reports a summary profile of respondents including (1) the size of organisation measured in terms of the total number of current employees, (2) geography measured by the country of headquarter, (3) industry sector of the organisation, and (4) the job function of the responding person. The data sample includes predominantly large enterprises, as almost 40 per cent of responses represent organisations with the total of employees exceeding 10,000.

About 85 per cent of the respondents work for organisations headquartered across Western Europe, in Switzerland and UK. Germany is the major country included in the sample covering almost 50 per cent of responses. Major industry sectors are the financial services (21%), transport and logistics (14%), high tech (10%), and automotive (9%). We observed a high rate of senior management participation (60 per cent) in the survey covering such positions as chief information officer (CIO), chief financial officer (CFO), chief executive officer (CEO), chief digital officer (CDO), board member, executive director, director, and business unit head. 40 per cent cover (senior) expert positions and roles.

The company name has been made optional to give the opportunity of an anonymous participation in the survey. We obtained 49 anonymous responses and 76 personalised responses including the contact details of the respondent and the company names. 76 personalised responses originate from 72 companies: 69 responses were one per company; 3 respondents answered for one company; finally, 2 companies were represented with 2 responses each. Since the over-representation of some companies in the sample is not of major concern here, we did not compensate the results for these multiple entries.

**Table 2:** Summary profile of respondents

Breakdown	Responses	Percentage
Sample size	125	100%
*Size in employees*		
Fewer than 500	33	26%
500–999	9	7%
1000–4999	21	17%
5000–9999	13	10%
10,000 or more	49	39%
*Countries of headquarter*		
Germany	63	50%
Switzerland	23	18%
France	15	12%
Belgium	3	2%
USA	3	2%
UK	2	2%
Other	16	13%
*Key industry sectors*		
Financial services (banking, insurance, and asset management)	26	21%
Transport and logistics	18	14%
High tech	12	10%
Automotive	11	9%
Manufacturing	8	6%
Consumer goods	7	6%
Communication, media and entertainment	7	6%
Energy and utilities	5	4%
Public sector	4	3%
Miscellaneous (healthcare, pharmaceuticals, chemicals and others)	27	22%
*Job function*		
IT manager	72	58%
Business manager	46	37%
Agile coach	7	6%

Note: percentages may not add up to 100 due to rounding

### Data analysis

Our data analysis approach has been primarily designed to examine correlations among the scores for six organisational dimensions introduced in the "Measures". To create comprehensive visuals and simplify interpretation of our results, we mapped the sample to a low-dimensional representation using a principal component analysis (PCA) method. We found no evidence for violation of normal distribution assumptions in the data set, therefore, we chose PCA as a simple and efficient method for dimensional reduction to generate aggregated features. We used normalised dimensional scores with mean $\mu = 0$ and standard deviation $\sigma = 1$.

The samples of the six organisational dimensions were clustered using the Fuzzy C-Means method (Bezdek 1981) with the fuzzifier-value of 1.1 and a selection of the number of clusters with the separation measure. Fuzzy C-means was chosen as a robust clustering method with a stable convergence behaviour towards similar solutions. Also, Fuzzy C-means can process gradual memberships of participants to the different clusters during the cluster generation. For the sake of simplicity, participants were assigned to the cluster by the highest membership score. The cluster distribution is visualised as a two-dimensional space of the aggregated features obtained from the PCA.

We used Qualtrics for the data collection and initial data processing purposes. For the PCA analysis and clustering process, we used the MATLAB toolbox SciXMiner (Mikut et al. 2017). Selected visuals and the interactive data room were developed with Tableau.

## Results

### Four profiles of agile organisations

Analysis of the dimensional scores reveals moderate positive correlations across all dimensions (see Table 3). The lowest correlation values were found between D2 (Organisation and structure) to D3 (Delivery and software development) and D2 to D4 (Product development) supporting the idea that the organisations either choose improving the technical space with agility or addressing organisational changes.

The PCA on normalised dimensional scores $D_\mathrm{{in}}$ reveals a mapping of the six-dimensional scores into a two-dimensional feature space using the aggregated features:

2 $$\begin{aligned} \mathrm{{AF}}_1 =&0.4151 \cdot D_{1N} + 0.3248 \cdot D_{2N} + 0.4222 \cdot D_{3N} + 0.3868 \cdot D_{4N} \nonumber \\&+ 0.4733 \cdot D_{5N} + 0.4128 \cdot D_{6N} \end{aligned}$$

3 $$\begin{aligned} \mathrm{{AF}}_2 =&0.3656 \cdot D_{1N} + 0.7510 \cdot D_{2N} -0.3131 \cdot D_{3N} -0.3805 \cdot D_{4N} \nonumber \\&-0.0355 \cdot D_{5N} -0.2412 \cdot D_{6N} \end{aligned}$$

4 $$\begin{aligned} D_\mathrm{{in}} =&\frac{D_i-\mu _i}{\sigma _i}. \end{aligned}$$

The first component is defined as the weighted mean of all dimensional scores and labeled as ’Aggregated Feature 1’ (AF1), exploiting the positive correlations across all dimensions. The second component focuses on the difference between the dimensional scores for D1 and D2 against other dimensional scores. We labeled this component as ’Aggregated Feature 2’ (AF2). These differences are highlighted by the positive and negative signs of the correlations between the second component and D1 and D2 vs. D3–D6 (see Table 3). First and second PCA components explain 49 per cent, respectively, 16.5 per cent of the total variance. The mean values and standard deviations for the normalisation of the dimensional scores are reported in Table 4.

**Table 3:** Pearson correlation coefficients for the dimensional scores and aggregated features

Feature	D1	D2	D3	D4	D5	D6
D1: Culture, values and leadership	1.00					
D2: Organisation and structure	0.49	1.00				
D3: Delivery and software development	0.40	0.19	1.00			
D4: Product development	0.41	0.12	0.43	1.00		
D5: Ways of working	0.41	0.41	0.52	0.42	1.00	
D6: Enterprise architecture	0.29	0.26	0.43	0.38	0.56	1.00
PCA1: Aggregated Feature 1	0.71	0.56	0.72	0.66	0.81	0.71
PCA2: Aggregated Feature 2	0.36	0.75	$-\,$0.31	$-\,$0.38	$-\,$0.04	$-\,$0.24

**Table 4:** Mean values and standard deviations for the dimensional scores

Feature	$\mu _i$	$\sigma _i$
D1: Culture, values and leadership	2.79	0.94
D2: Organisation and structure	2.86	1.01
D3: Delivery and software development	2.90	1.06
D4: Product development	3.14	1.18
D5: Ways of working	2.61	0.98
D6: Enterprise architecture	2.58	1.00

**Fig. 1:**
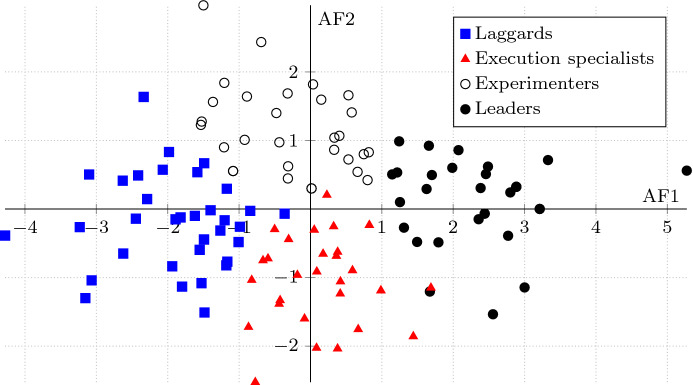
Scatter plot with Aggregated Features 1 and 2

Figure 1 shows the values of the Aggregated Features 1 and 2 as a scatter plot. The dots represent values of Aggregated Feature 1 (x-axis) and Aggregated Feature 2 (y-axis). The position of each dot on the horizontal and vertical axes indicate each individual response in the survey. The visual analysis of the scatter plot leads to impression that the clusters have blurred boundaries. However, the visual representation appeared to be useful in discussing the positioning of individual responses relative to its peers by industry, geography or company size.

Cluster analysis based on six organisational dimensions resulted in four clusters which we interpreted as four empirical profiles of agile organisations. Table 5 describes these four clusters by using cluster mean values of six-dimensional scores. Based on the interpretation of the dimensional scores, we labeled the clusters as laggards (lowest scores across all dimensions), execution specialists (high scores for delivery and software development, other scores around sample average), experimenters (high scores for organisation and structure, above average score for culture, values and leadership, other scores around sample average), and leaders (highest scores across all dimensions).

**Table 5:** Breakdown of dimensional scores by cluster

	Laggards	Experimenters	Execution specialists	Leaders
Cluster size	35	30	30	30
D1: Culture, values and leadership	2.1	2.9	2.4	3.8
D2: Organisation and structure	2.0	3.7	2.2	3.7
D3: Delivery and software development	2.3	2.2	3.1	4.1
D4: Product development	2.4	2.5	3.7	4.1
D5: Ways of working	1.6	2.6	2.7	3.6
D6: Enterprise architecture	1.7	2.2	3.1	3.4
Overall level of organisational agility	2.0	2.7	2.9	3.8

*Laggards* (*n* = 35): Agile practices and DevOps elements are piloted across organisation as isolated spots, especially within the IT and R&D departments. No enterprise-wide agile culture established. Overall organisational structure remains unchanged. Project-orientated thinking prevails. No product orientation.

*Experimenters* (*n* = 30): High scores across the dimensions relating to corporate culture, values, leadership and organisation. Novel organisational forms, cross-functional collaboration and agile processes are on top of the agenda. Clear focus on people rather than technical maturity. Organisations within this clusters are more likely to start agile transformations on the business side or from the people’s perspective.

*Execution specialists* (*n* = 30): Agile tools and practices are well established and support the agile delivery model. Clear product orientation and working along the value streams are institutionalised. Structure, roles and responsibilities remain Tayloristic. The tipping point in terms of culture, values and organisation is not reached.

*Leaders* (*n* = 30): Consistently high scores across all dimensions. High level of customer integration based on agile delivery model. Tipping point across culture, values and organisation clearly reached. Agile ways of working are dominating across the organisation. Agile delivery model includes product orientation, short time-to-market and frequent customer feedback cycles.

### Design of agile transformation initiatives

#### Affected organisational dimensions

The survey asked participants to point out key organisational dimensions affected most by the transformation efforts within in their organisations (see Table 6). Respondents perceive that project management, delivery and software development, processes, and product development are impacted most by agile transformation initiatives. However, while the laggards, experimenters and execution specialists agree on project management being impacted most, the leaders direct attention to processes and product management instead. Also, leaders have higher number of responses relating to culture and values as well as the goal setting approach.

Table 6 further reports average numbers of selected dimensions by response. On average, participants have selected 3.7 dimensions. While the experimenters and execution specialists hover around the sample average, laggards have a lower value of 3.3. The leaders stand out with 4.4 supporting the idea that this cluster seeks a more holistic approach with a greater organisational reach compared to another clusters. In another words, companies with above-average levels of organisational agility tend to design their agile transformation initiatives with a greater organisational reach by tacking a larger number of organisational dimensions.

**Table 6:** Which dimensions of your organisation have been affected most by agile transformation [count responses]

Dimension	Laggards	Experimenters	Execution specialists	Leaders	Total
Project management	18	18	20	20	76
Delivery and software development	16	16	15	16	63
Processes	15	14	13	20	62
Product development	13	9	10	15	62
Leadership	11	10	9	11	41
Culture and values	7	10	7	13	37
Organisational structure	4	13	9	9	35
Software maintenance	9	4	5	7	25
Goal setting	3	4	7	10	24
Demand management	4	6	4	4	18
Architecture	5	3	4	3	15
Governance	5	2	1	3	11
Portfolio management	4	2	4	1	11
Average number of dimensions per response	3.26	3.70	3.60	4.40	3.72

#### Reported share of agile projects in the project portfolio

Table 7 summarises reported shares of agile projects in the project portfolios of the respondents. Our results confirm that companies enacting higher levels of organisational agility have higher share of agile projects in their portfolios. Indeed, 60 per cent of the leaders report the share of agile projects of 60 per cent or more. However, our data do not allow us to confirm the underlying causal mechanisms between organisational agility and share of agile projects. Indeed, high share of agile projects might lead to higher levels of organisational agility. Or vice versa, high organisational agility might be a cause to higher shares of agile projects. Further research can more clearly delineate the underlying reasons of this relationship.

**Table 7:** Reported share of agile projects in the project portfolio by cluster

	< 20%	20–40%	40–60%	> 60%	Total
Laggards	19	11	–	5	35
Experimenters	6	10	7	7	30
Execution specialists	13	10	2	5	30
Leaders	3	6	3	18	30

#### Reported length of experience with agile methods

Table 8 summarises reported length of experience with agile methods by cluster. The vast majority of participants confirmed having experience with agile methods of less than 4 years: laggards (71%), experimenters (83%), execution specialists (76%), and leaders (50%). Leaders seem to have longer experience with agile methods compared to another clusters supporting the idea that greater organisational agility comes along with experience. However, our data do not allow us to conclude that longer experience does lead to greater organisational agility or vice versa.

**Table 8:** Length of experience with agile practices by cluster

	< 2 years	2–4 years	4–6 years	> 6 years	Total
Laggards	16	9	5	5	35
Experimenters	11	14	3	2	30
Execution specialists	14	9	3	4	30
Leaders	5	10	6	9	30

#### Perceived level of adoption of agile methods relative to competition

The survey asked participants to report the level of adoption of agile methods relative to competitors (see Table 9). The perception of respondents varies significantly by cluster. The laggards predominantly consider being at same level or worse compared to the competition (86%). Only 14 per cent of the laggards rate themselves better than the competition in terms of using agile methods. 40 per cent of the execution specialists and 43 per cent of the experimenters report having a higher level of adoption compared to the competition. Finally, 70 per cent of the leaders feel having a higher level of adoption. The general perception seems to be realistic: organisations exhibiting lower levels of organisational agility tend to grade themselves below the competition and vice versa. The respondents with higher levels of organisational agility report more confidence and satisfaction from using agile methods. We have not found any statistically significant deviation by industry, geography or company size.

**Table 9:** Perceived level of adoption of agile practices relative to competitors [count responses]

	Much better	Somewhat better	About the same	Somewhat worse	Much worse	Total
Laggards	–	5	14	10	6	35
Experimenters	3	10	13	3	1	30
Execution specialists	3	9	9	7	2	30
Leaders	9	12	7	2	–	30

#### Relation to digitalisation initiatives

Table 10 summarises the usage of digitalisation initiatives by organisations from our data set. Multiple answers were allowed. Digitalisation projects and initiatives are at the top of the agenda for all four groups, followed by digital strategy, except for the laggards focusing on automating business processes instead. Furthermore, the vast majority of respondents direct attention to developing digital products and services; however, the leaders seem to connect those initiatives with developing novel digital business models.

Furthermore, following current discussion on the role of a chief digital officer (Wladawsky-Berger 2012; Tumbas et al. 2018; Haffke et al. 2016), our data confirm low level of adoption for this role. Clusters with higher level of organisational agility seem to avoid this role supporting the idea that organisations are more effective when digital competence is incorporated into the DNA of the entire organisation rather than concentrated in one particular unit.

**Table 10:** Current use of digital initiatives [count responses], LAG: laggards, EXP: experimenters, ESP: execution specialists, LEA: leaders

	LAG	EXP	ESP	LEA	Total
Digital transformation projects and initiatives	27	23	22	23	95
Digital strategy	15	23	19	20	77
Digital products and services	19	15	14	19	67
Automated business processes	16	14	14	16	60
Digital business models	11	14	8	17	50
Chief digital officer	12	10	7	5	34
Digital factory	8	9	8	3	28
None from above	3	2	2	1	8
Average number or initiatives per response	1.8	3.7	3.1	3.5	3.4

Table 10 further reports average numbers of digital transformation initiatives by cluster. While leaders, experimenters and execution specialists hover around the average of 3.4 initiatives, the laggards report using less than two initiatives. With the average value of 3.7, the experimenters are slightly above average. The results can be interpreted in favour of the idea what level of organisational agility is connected with overall level of digitalisation. However, further research is needed to confirm this idea. Also, our data set does not include any hints on causation: does higher level of organisational agility lead to a higher level of digitalisation or vice versa.

### Agile at scale

Table 11 provides a summary statistics for the current use of agile scaling frameworks. Multiple answers were allowed. 76 per cent of respondents have confirmed deploying agile scaling frameworks. Scrum of Scrums and Lean Management are leading the list with 44 and 31 responses. Another 31 respondents have confirmed using an internally developed framework.

**Table 11:** Usage of agile scaling frameworks (breakdown responses by cluster)

Framework	Laggards	Experimenters	Execution specialists	Leaders	Total
Scrum of Scrums	7	19	4	14	44
Lean management	3	13	3	12	31
Internally created method	6	9	4	12	31
SAFe	4	8	2	9	23
LeSS	2	2	2	7	13
Agile portfolio management	–	3	3	7	13
Disciplined agile delivery	1	1	–	3	5
Other	–	1	–	2	3
We do not scale agile methods	20	–	18	3	41

Table 12 presents our results relating to the number of agile scaling frameworks in use at a time. The vast majority (62 responses) uses 1 or 2 agile scaling frameworks. Another 22 respondents revealed using 3 or more frameworks simultaneously. The sample average number of agile scaling frameworks equals 1.30 supporting the idea that, on average, the respondents tend to deploy one or two agile scaling framework at a time. However, the averages vary significantly by cluster. The laggards and execution specialists have the lowest average of 0.66 and 0.60, respectively, revealing that only every second respondent within those clusters uses agile scaling frameworks. The averages rise up to 2.20 for the leaders and 1.87 for the experimenters showing that those groups tend to simultaneously deploy two agile scaling frameworks.

**Table 12:** Number of agile scaling frameworks in current use

	Number of frameworks in use							
	0	1	2	3	4	5	Checksum	Average
Laggards	20	9	4	2	–	–	35	0.66
Execution specialists	18	6	6	–	–	–	30	0.60
Experimenters	–	12	11	6	1	–	30	1.87
Leaders	3	8	6	9	1	3	30	2.20
Total	41	35	27	17	2	3	125	1.30

### Business agility

Table 13 highlights current perception of respondents with regard to the adoption of agile methods across individual organisational functions. The respondents have been asked to select up to three most agile and least agile functions within their organisations. The number of responses is reported in the corresponding column of the table. All items are sorted in descending order by the value of the first column (most agile). The respondents seem to agree that information technology and product development behave in agile manner in their organisations. Indeed, 65 per cent of respondents confirm that IT is the most agile function, while only 15 per cent see IT as non-agile. Similarly, 54 per cent rate product development as agile, while only 6 per cent consider this function as non-agile.

Supporting functions such as human resources, corporate finance, general administration, as well as legal service, risk management and compliance are seen by the majority of the respondents as the least agile functions within their organisations. Only 4 per cent of respondents rate human resources, finance and administration as agile, while roughly 70 per cent confirm those functions being non-agile in their organisations. Also, 24 per cent respondents rate the line management within their organisations as non-agile supporting the idea that there is still a need in facilitating agile leadership style and behaviours across organisations.

Respondents seem to be indifferent whether marketing and communications as well as customer service and support are currently more agile or non-agile. This observation may indicate that there is a lot of transition going on within those functions and there is no clear view of current results across organisations.

**Table 13:** Respondents’ perception of the most and least agile organisational functions [count responses]

Organisational function	Most agile	Least agile
Information technology	81	19
Product development	68	7
Research	48	10
Production and operations	35	26
Marketing and communications	21	23
Customer service and support	19	18
Sales	13	23
HR, finance and administration	5	87
Legal, risk and compliance	4	71
Line management	2	30

## Discussion

We address the relationship between technical excellence, agile organisational design and agility. While the practice literature has encouraged managers to expect that organisational design changes enacted during agile transformation secure organisational agility, our findings suggest a more subtle relationship between technical excellence and agile organisational design. Four identified profiles (leaders, experimenters, execution specialists, and laggards) suggest that organisational agility can be built upon technical excellence (execution specialists) and agile organisational design (experimenters). However, combined in an intelligent way, both factors will create a consistent profile (leaders).

When designing and implementing agile transformations, managing multiple organisational dimensions is critical for success. Understanding how the leaders achieve organisational agility requires a nuanced appreciation of the link between agile organisational design, technical excellence, corporate culture and leadership styles. We argued that attempts to achieve greater organisational agility are associated with building more agile business processes rather than focusing on project work. Employees with agile mindset would voluntarily choose agile ways of working and shift project work into agile modes; however, the management has to establish the underlying framework of agile and lean processes to enable employees work effectively. 74 per cent of the leaders agree that processes such as planning, budgeting and resource allocation are flexible enough to adjust to changing priorities, compared to 9 per cent of laggards. This finding runs counter to prescriptive literature and general managerial practice that advocate a greater reliance on agile project work to enable organisational agility. Though our data do not allow us to confirm underlying casual mechanisms, it is possible than agile business processes enable organisational to act in a more agile manner. Further research can more clearly delineate the reasons for this relationship. Technical excellence is mainly achieved through continuous delivery, deployment and integration, test automation and decoupled architectures. Focusing on product management rather than IT management allows the leaders to consider the entire value chain by looking into activities how new products are created and existing products are modified.

When companies focus on designing agile transformations, managers actively seek to extend organisation by using agile scaling frameworks. In this context, scaling agile methods seem to be positively correlated with the level of organisational agility. Experimenters and leaders reported to use around two frameworks simultaneously. This finding suggests that the advice in the practice literature on agile transformation design as a process of scaling agile practices is accurate. Companies that follow the traditional transformation approach, assuming that agile practices should be implemented on a team level first, may find themselves unable to scale agile practices up to the organisation-wide level. This result is more inline with the idea of designing agile transformation closely with an introduction of an agile scaling framework.

The respondents perceive that length of experience with agile practices is positively correlated with the level of organisational agility. Our findings suggest that a time frame of around 2 years is needed to achieve best possible impact from an agile transformation initiative. While managers seem to focus on increasing agility within IT, product development and research functions, a significant element of achieving agility stems from improving supporting functions. The respondents confirm supporting functions such as HR, finance, administration, legal, risk and compliance are currently run in a non-agile manner.

Based on the findings of our research, we suggest the following approach to setup an agile transformation initiative:

1. Start with the assessment of your organisation in terms of belonging to one of the clusters by applying the questionnaire from "Survey questions".
2. Set the aspiration level of your organisation in the form of goals and objectives. For instance, a laggard can set the aspiration to become an execution specialist or even a leader.
3. Set the course of action:
  For the laggards: start with creating more agile business processes and simultaneously imposing agile mindset across the organisation.
  For the execution specialists: improve the organisational form, e.g. by institutionalising new agile roles and responsibilities, creating cross-functional organisational units etc.
  For the experimenters: implement and roll-out end-to-end business processes and improve technical foundations, e.g. by creating a decoupled architecture, automating tests etc.
  For the leaders: implement the continuous improvement mechanisms and build greater business agility by transforming support functions, e.g. HR, finance, administration and others.
4. Consider timing-related questions:
  Agile scaling frameworks require a certain level of organisational agility and, therefore, are best to implement for experimenters, execution specialists and leaders. The best practice is also to combine individual elements of the frameworks into an internally created method reflecting all specifics of the organisation.
  For the laggards: when setting up an agile transformation programme, the organisation should plan for a time frame of around 2 years. Individual projects and initiatives might have shorter time frames; however, the greater, long lasting impact within the organisation is achieved after two years. Organisations belonging to other clusters might need shorter time frames.
5. Monitor the progress against the goals and objectives set in step 2. The assessment mentioned in step 1 can be applied repeatedly (e.g. semi-annually, annually) to quantify the status quo and re-iterate the process.

The assessment approach and target setting mechanisms mentioned in steps 1, 2 and 5 have to be further detailed. This requires further research including longitudinal studies for the development of agile companies over time.

### Limitations

This survey has potential limitations. This research is limited to certain geography as the sample covers predominantly the European area and UK. Also, it is limited to certain industry branches and sectors, as the sample does not cover all industry branches and sectors, e.g. non-profit organisations.

The number of research questions included into the questionnaires has been limited to avoid the survey fatigue. Therefore, further research questions that might appear relevant in this context have not been investigated (cf. "Outlook and further directions" for more examples).

As the survey has been non-compulsory, the sample has anonymous responses. The findings have no link to individual companies or organisations.

The subject of research lies within a rapidly changing environment. Therefore, the most recent developments raised within the last 6–9 months are not covered (e.g. link between agility and resiliency in the context of the COVID-19 outbreak).

### Outlook and further directions

Despite of the fact that major relevant perspectives of the organisational agility have been covered by this survey, further research questions might be of essence and are worth being investigated.

When discussing the survey results among the academic peers and survey participants, we have come to realise that the academic and industry are interested in further investigating the financial impact from introducing agile methods and tools. Here, the research related to relevant financial metrics and the methodology to build a business case might be an important future direction (Yauch 2011; Pulakos et al. 2019).

In light of recent events and the outbreak of the Corona virus, the relationship between resilience and agility should be better understood, see (Batra 2020). Here, the development of metrics measuring the resilience and describing the relation to the organisational agility might be of relevance.

The usage of agile tools and methodologies within the Human Resource departments is considered to be one of novel research directions. Agile tools and methods might help organisations attract, retain, and develop talent, and transform internal HR processes towards agility. As an contribution to this strategy, Harsch and Festing explore the role of dynamic talent management capabilities in the organisational agility (Harsch and Festing 2020).

## Annex

### Survey questions

1. How long has your organisation been using agile methods: fewer that 2, 2–4, 4–6, 6 years or more.
2. What is the share of agile projects in your IT project portfolio: fewer that 20, 20–40, 40–60, 60 per cent or more.
3. How effective would you say your organisation’s agile transformation efforts have been to date: 0 = not effective at all, 10 = extremely effective (integer scale).
4. Which dimensions of your organisation have been affected most by agile transformation? Please select up to five: organisational structure, processes, leadership, culture and values, goal setting, delivery and software development, software maintenance, product development, architecture, project management, demand management, governance, portfolio management.
5. Please respond to each item in terms of how does it apply to your organisation (definitely true, probably true, neither true nor false, probably false, definitely false):
  - The management demonstrates leadership styles building upon employee empowerment, cross-functional collaboration and short feedback cycles.
  - Agile values and principles are well known across the organisation.
  - The organisation has established a positive failure attitude and embraces risk taking.
  - Employees across the organisation have been equipped with substantial decision rights and exercise those rights.
  - The management team has initiated organisational changes to further facilitate agile transformation.
  - Project teams are staffed in a cross-functional manner and engage in cross-functional collaboration.
  - The organisation has implemented new agile organisational models, e.g. value streams, virtual organisations.
6. In your view, which areas of your organisation are most/least agile? Please select up to three for each column: IT, product development, research, production and operations, customer service and support, marketing and communications, sales, HR/finance/administration, legal/risk/compliance, line management.
7. Please rate the extent to which you agree with each of the following statements (strongly disagree, somewhat disagree, neither disagree nor agree, somewhat agree, strongly agree):
  - In my organisation, tests are run in an automated manner and executed throughout the implementation phase.
  - Continuous delivery, deployment and integration enable my organisation to deliver changes more frequently and reliably.
  - My organisation has the ability to continuously incorporate customer feedback into the product development.
  - Agile methods and tools are used for project-independent activities, e.g. maintenance, incident tracking, environment teams, value stream teams.
  - When starting a new project, I can refer to decision criteria in my organisation on where and how to use agile methods.
  - Planning, budgeting and resource allocation processes are flexible enough to fluidly adjust to changes in my organisation’s priorities.
  - My organisation has established architecture principles supporting agile development through collaboration, emergent design, and design simplicity.
  - Enterprise architecture is organisationally embedded into agile team structures.
8. How would you rate your organisation’s level of agile adoption relative to your competitors? (Much better, somewhat better, about the same, somewhat worse, much worse).
9. Which of the following agile scaling frameworks do you use in your organisation? Please select all that apply: Scaled Agile Framework (SAFe), Scrum of Scrums, Lean Management, Agile Portfolio Management, Large-Scale Scrum (LeSS), Disciplined Agile Delivery (DAD), Recipes for Agile Governance in the Enterprise (RAGE), Nexus, internally created method, we do not scale agile methods.
10. Which of the following can be found within your organisation? Please select all that apply: automated business processes, digital strategy, digital transformation projects and initiatives, digital products and services, digital business models, digital factory, chief digital officer, none from above.

*These last four questions are for classification purposes only. Please proceed.*

1. How many individuals does your organisation employ (all locations): fewer than 500, 500–999, 1,000–4,999, 5,000–9,999, 10,000 or more.
2. Which of the following best describes the industry sector in which you work: automotive, insurance, financial services (bank, asset management) excl. insurance, consumer goods, public sector, life sciences (pharmaceuticals, biotechnology), chemicals and materials, communications/media/entertainment, high tech, healthcare, energy and utilities, transport and logistics, other (free text).
3. What is your main functional roles: business manager, IT manager, agile coach.
4. Where is your organisation headquartered: Germany, France, Austria, Switzerland, UK, other (free text).
5. Do you want us to share the survey results report with you? We will ask you to provide your contact information (name, position, company and email address). As soon as we have completed the survey, we will send you the download link to the survey results on your email address. By clicking on ”I agree”, you give consent to the processing of your contact information. Your consent is entirely voluntary and can be withdrawn at any time, without giving of any reasons and with effect for the future. To withdraw your consent, please contact datenschutz@kobaltblau.com.
6. Please provide your contact information for receiving the survey results report: name, position, company, email address (shown only if the respondent has given consent to data processing in the previous question).

## Data Availability

All collected raw data and responses cannot be made publicly available to avoid any possible disclosure of sensitive information relating to the study participants. The study participants have been advised that provided responses will remain confidential at any circumstance.

## Footnotes

1 Reflect recent developments in the area of organisational theory and design in response to the digitalisation and the rise of novel digital, data-based and other business models.

2 Can be seen as a set of organisational rules, principles and workflow patterns intended to guide an organisation in scaling agile practices.

## Declarations

### Preprint

A preprint version of this paper was published by https://arxiv.org/abs/2006.00048.
